# Associations between haemoglobin-to-red cell distribution width ratio and mortality in intracerebral haemorrhage: a population-based analysis of the MIMIC-IV database

**DOI:** 10.7189/jogh.16.04065

**Published:** 2026-04-17

**Authors:** Bo-An Chen, Shin-Nan Lin, Kuo-Chen Wei, Yi-Ying Hsieh, Jiun-Lin Yan, Mao-Yu Chen, Yi-Hsien Kuo, Ying-Yun Chen, Pin-Yuan Chen

**Affiliations:** 1 Department of Neurosurgery, Chang Gung Memorial Hospital, Linkou, Taiwan; 2 Department of Medical Imaging and Intervention, Linkou Chang Gung Memorial Hospital, College of Medicine, Chang Gung University, Taoyuan City, Taiwan; 3 Department of Neurosurgery, Jen-Ai Hospital, Taichung, Taiwan; 4 Department of Neurosurgery, Chang Gung Memorial Hospital, Keelung, Taiwan; 5 College of Medicine, Chang Gung University, Taoyuan, Taiwan

## Abstract

**Background:**

Intracerebral haemorrhage (ICH) is a leading cause of mortality and morbidity worldwide. Identifying early prognostic biomarkers is crucial to optimise clinical management of critically ill patients with ICH. The haemoglobin-to-red cell distribution width ratio (HRR) has recently emerged as a potential predictor in various critical illnesses, but its role in ICH remains unclear. This study aimed to evaluate associations between HRR and mortality in patients with ICH.

**Methods:**

This retrospective cohort study included the data of adults (≥18 years) extracted from the Medical Information Mart for Intensive Care-IV (MIMIC-IV) database (version 1). HRR was calculated from the first available haemoglobin and RDW measurements within one day of each other. Patients were categorised into HRR quartiles. The primary outcomes were 28-day and 1-year all-cause mortality. Associations were assessed using Cox proportional hazards models.

**Results:**

The data of 1915 patients (mean age 67.5 years) were analysed retrospectively. After adjusting for possible confounders, compared with the lowest HRR quartile (Q1), patients in Q3 and Q4 had significantly lower 28-day mortality (Q3: adjusted hazard ratio (aHR) = 0.72; 95% CI = 0.54, 0.97; Q4: aHR = 0.67; 95% CI = 0.49, 0.90). Similarly, higher HRR quartiles were associated with reduced 1-year mortality risk (Q3: aHR = 0.64; 95% CI = 0.49, 0.84; Q4: aHR = 0.56; 95% CI = 0.42, 0.75).

**Conclusions:**

Lower HRR at admission is independently associated with higher short-term and long-term mortality in ICU patients with ICH. HRR may serve as a candidate prognostic biomarker for early risk stratification in this high-risk population. Further prospective studies are warranted to confirm these findings.

Intracerebral haemorrhage (ICH) represents a significant public health concern due to its high morbidity and mortality rates. For example, ICH accounts for approximately 15–30% of all strokes [1], with an annual global incidence of about 3.4 million new cases [2]. Only about one-quarter of ICH patients achieve functional independence by three months [3]. This poor prognosis underscores the need to improve risk stratification and to find early prognostic biomarkers for ICH, particularly for critically ill patients in intensive care units (ICUs). Identifying reliable predictors of mortality would likely support clinical decision-making and resource allocation for this vulnerable population.

Recent studies have shown that red blood cell (RBC) morphology and oxygen-carrying capacity can be potential prognostic markers in clinical outcomes [4,5]. Specifically, red cell distribution width (RDW), a measure of anisocytosis (variations in RBC size), has been associated with systemic inflammation and adverse outcomes in various cardiovascular and neurological diseases such as heart failure or stroke [4,5]. In ICH, elevated RDW on admission is an independent predictor of higher mortality. Pinho et al. [4] reported 3-fold increased odds of 30-day mortality in patients in the highest RDW quartile compared to those with lower RDW, after adjusting for other risk factors. Similarly, low haemoglobin (Hb) levels and the presence of anaemia have demonstrated prognostic significance in haemorrhagic stroke. Anaemia may exacerbate brain injury by reducing oxygen delivery and increasing ischemic vulnerability in the peri-haematomal region. Lower admission haemoglobin has been shown to correlate with greater mortality and disability after ICH[6]. Even mild to moderate anaemia has been linked to significantly higher 90-day mortality risk in patients with ICH [1]. These previous findings support the biological plausibility that both impaired oxygen-carrying capacity and underlying inflammatory stress (reflected by RDW) adversely affect outcomes in acute brain haemorrhage.

Given the individual prognostic importance of haemoglobin and RDW, the haemoglobin-to-RDW ratio (HRR) has emerged as a novel composite biomarker that captures both parameters simultaneously [5]. Growing evidence demonstrates that HRR, available as a simple, routine blood test, is a robust predictor of outcomes across multiple or severe conditions. In critically ill sepsis patients, for example, a higher HRR at ICU admission has been independently associated with improved survival: Zhong et al. [7] found that HRR was inversely associated with 30-day and 1-year mortality in a large ICU cohort, the Medical Information Mart for Intensive Care-IV (MIMIC-IV) database. HRR outperformed either Hb or RDW alone as a predictor for mortality among patients admitted with stroke [8]. Likewise, in patients with acute pancreatitis, those in the lowest HRR quartile had a significantly greater 30-day mortality risk, and incorporating admission HRR results improved the accuracy of mortality risk models beyond conventional prognostic scoring systems such as Sequential Organ Failure Assessment (SOFA) and Bedside Index of Severity (BIS) in Acute Pancreatitis [9]. In acute ischemic stroke, results of preliminary studies suggest similar trends: a low HRR ratio has been associated with higher mortality after thrombolysis [10], and a reduced HRR measured 24 hours after mechanical thrombectomy was independently associated with higher odds of poor outcomes and death at 90 days [11].

Despite the evidence outlined above, data on HRR in the context of intracerebral haemorrhage is lacking. To the best of our knowledge, no prior study has specifically evaluated the prognostic value of HRR in patients with ICH, particularly those in critical care settings. Existing studies on HRR have focused on ischemic stroke or mixed cohorts, and none have examined this biomarker in an exclusively ICH population. We hypothesised that a lower HRR on admission would be an independent predictor of higher mortality in ICH. Therefore, this study aimed to evaluate associations between HRR and mortality in ICU patients with ICH.

## METHODS

### Data source

This population-based retrospective cohort study extracted all patient data from the MIMIC-IV database (version 1.0), a publicly available and de-identified clinical data set developed by the Massachusetts Institute of Technology (MIT) and Beth Israel Deaconess Medical Center (BIDMC). MIMIC-IV contains detailed clinical data of over 60 000 ICU admissions and more than 200 000 emergency department (ED) visits at BIDMC between 2008 and 2019, including patients’ demographic and clinical characteristics (*i.e.* vital signs, laboratory results, procedures, medications, and clinical notes). Institutional Review Board (IRB) approvals were obtained in advance (Massachusetts Institute of Technology, No. 0403000206; Beth Israel Deaconess Medical Center, Protocol 2001P001699). MIMIC-IV also offers several enhancements over its predecessor, including a streamlined structure, expanded data elements, and improved usability. The database complies with the Health Insurance Portability and Accountability Act (HIPAA) Safe Harbor provision, with rigorous de-identification procedures such as date shifting and removal of protected health information from structured and free-text data. Access to MIMIC-IV requires completion of the Collaborative Institutional Training Initiative (CITI) programme and a data use agreement. We successfully completed the CITI certification, granting us permission to access and extract data for analysis.

### Ethics statement

This study was conducted in accordance with the ethical standards of the institutional research committees and the principles outlined in the Declaration of Helsinki. Also, as noted above, IRB approvals were obtained by MIMIC, including from MIT (No. 0403000206) and BIDMC (No. 2001P001699). Because this study involved only secondary analysis of de-identified patient data from a publicly available database with no direct patient involvement or identifiable private information collected, the requirement for informed consent was waived.

### Patients

Patients were selected from the MIMIC-IV database based on specific inclusion and exclusion criteria. Adults aged 18 years or older who were admitted to the ICU for the first time during their initial hospitalisation for ICH were included. ICH was identified using ICD-9-CM code 431 or ICD-10-CM codes I610, I611, I612, I613, I614, I615, I616, I618, and I619. The index date was defined as the date of the patient’s first ICU admission during the initial hospitalisation for ICH.

Patients whose total ICU length of stay (LOS) during the index admission was less than 24 hours, or who were missing complete data for HRR calculation, or whose first available HRR measurement was obtained more than 10 days after ICU admission were excluded. Patients with an ICU length of stay less than 24 hours were excluded to minimise the inclusion of patients admitted for short-term postoperative monitoring or those who died or were discharged within the first 24 hours, which could introduce immortal time bias and dilute the association between the exposure of interest and outcomes.

### Study outcomes

The primary outcomes of this study were 28-day mortality and 1-year mortality following ICU admission. For 28-day mortality, all-cause death occurring within 28 days after the index ICU admission was defined as the outcome event. Mortality status was determined using the date of death (DOD) recorded in the patients file of the MIMIC-IV database. Patients who died within 28 days of admission were classified as non-survivors, and those who survived beyond 28 days were classified as survivors. For 1-year mortality, all-cause death occurring within 365 days after ICU admission was considered the event of interest. Patients were followed for exactly 365 days from the index ICU admission. Patients without a recorded death date within this period were considered to be alive and were censored at 365 days.

### Exposure variable

The exposure variable in this study was the HRR. Haemoglobin and RDW values were extracted from the LABEVENTS file of the MIMIC-IV database. Haemoglobin measurements were identified using ITEMIDs 50811, 51222, and 51645, and RDW measurements were identified using ITEMID 51277. For each patient, the first available Hb and RDW measurements obtained after hospital admission were used, including values collected during the ED stay or hospitalisation. Because Hb and RDW results were not always available on the same day, measurements were paired if their recorded result times were within one day of each other. Among eligible pairs, the set of measurements closest to the index ICU admission date was selected. HRR was calculated by dividing the Hb concentration (g/dL) by the RDW percentage (%). For the purpose of further analysis of data, patients were subsequently categorised into HRR quartiles.

### Covariates

Covariates were grouped into five categories: demographic characteristics, clinical characteristics, comorbidities, laboratory data, and standard scoring schemes. Demographic variables included age (categorised as 18–39, 40–59, 60–79, and ≥80 years), sex (female or male), and race/ethnicity (White, Black, Hispanic/Latino, Asian, or Other). Clinical, anthropometric and lifestyle characteristics included body mass index (BMI, kg/m^2^), history of tobacco use, use of anticoagulants, vital signs recorded closest to the index ICU admission (systolic blood pressure (SBP, mmHg), diastolic blood pressure (DBP, mmHg), heart rate (beats per minute), respiratory rate (inspirations per minute), peripheral oxygen saturation (SpO_2_, %), mean arterial pressure (MAP, mmHg), body temperature (°C)), and Glasgow Coma Scale (GCS) scores. Comorbidities included diabetes mellitus (DM), chronic kidney disease (CKD), aneurysm, hypertension, atrial fibrillation, chronic heart failure (CHF), coronary heart disease (CHD), chronic obstructive pulmonary disease (COPD), chronic liver disease, and dementia. The presence of infection during hospitalisation was also recorded. Laboratory data included glucose (mg/dL), haemoglobin A1c (HbA1c, %), ferritin (ng/mL), serum albumin (g/dL), white blood cell (WBC) count (K/μL), red blood cell (RBC) count (M/μL), red cell distribution width (RDW, %), platelet count (K/μL), lymphocyte count (K/μL), haemoglobin (g/dL), blood urea nitrogen (BUN, mg/dL), serum creatinine (mg/dL), serum sodium (mEq/L), serum potassium (mEq/L), International Normalized Ratio (INR), and C-reactive protein (CRP, mg/L). Standard scoring schemes used to evaluate illness severity included the Oxford Acute Severity of Illness Score (OASIS), SOFA score, Simplified Acute Physiology Score II (SAPS II), and the Charlson Comorbidity Index (CCI). Additionally, ICU LOS and hospital LOS were collected as clinical outcome variables.

### Statistical analysis

This study used data from the MIMIC-IV database, v1.0. Descriptive statistics for the study population were presented as mean ± standard deviation or median with interquartile ranges (IQRs) for continuous variables, and as frequencies with proportions for categorical variables. HRR was divided into four equal-sized groups, *i.e.* quartiles, to compare the different quartiles from low to high. One-way analysis of variance (ANOVA) or Kruskal-Wallis tests for skewed data were applied for continuous variables, while χ^2^ tests or Fisher exact tests were used for categorical variables.

Prior to model adjustment, variability analysis was performed for continuous covariates to ensure the absence of multicollinearity. The proportional hazards assumption for HRR and mortality was then examined with Schoenfeld residuals. After confirming that the assumption was satisfied, associations between HRR quartiles and short-term (28-day) and long-term (1-year) mortality were analysed using Cox proportional hazards models. Multivariable models were employed to estimate the hazard ratios (adjusted hazard ratios) associated with HRR quartiles, adjusting for covariates identified by clinical experts as relevant, including anticoagulants, Charlson Comorbidity Index (CCI), International Normalized Ratio, infection, and creatinine. Kaplan-Meier survival analysis was employed to estimate the time-to-event distribution and to visualise the survival probability over time among the study cohorts. Additionally, subgroup analyses were performed to examine whether the mortality risk associated with HRR quartiles differed between patients with and without CHD, and among those using anticoagulants and those not. Sensitivity analyses were employed to present the associations between haemoglobin quartiles, RDW quartiles, and both 28-day and 1-year mortality, which allowed direct comparison of the prognostic utility of HRR. All statistical analyses were conducted using SAS software version 9.4 (SAS Institute Inc., Cary, NC, USA), and a two-sided *P*-value of <0.05 was considered statistically significant.

## RESULTS

### Study population selection

**Figure 1:**
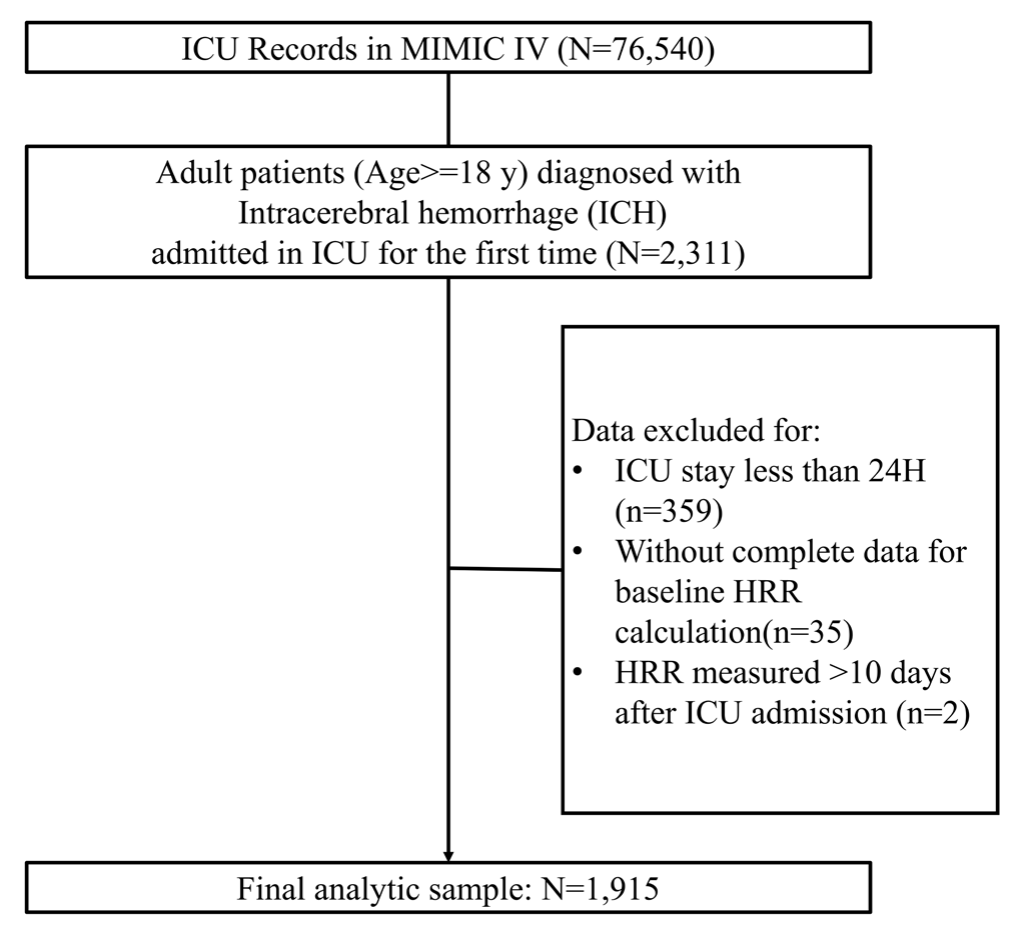
Flowchart of patient selection.

A total of 2311 adult patients (age> = 18 years) diagnosed with ICH admitted into ICU for the first time. Patients who stayed in ICU less than 24 hours (n = 359) or lacked baseline data for HRR calculation (n = 37) were excluded. Finally, after exclusions, the data of 1915 patients were included as the analytic sample (**Figure 1**).

### Characteristics of the study population

Mean age for the entire study population was 67.5 years, males 54.6%. The majority of the cohort (60.8%) was white. The most common comorbidity (46 was CHD (46.1%), followed by hypertension (40.4%). Majority of the cohort (60.8%) was white. Among these patients, the median OASIS Score was 33 (IQR = 27–39), SOFA score 3 (IQR = 2–5), and SAPS II score (IQR = 26–40) (**Table 1**).

**Table 1:** Patients’ demographic and clinical characteristics compared between HRR quartiles

Characteristics	All (n = 1915)	HRR				*P-*value
		Q1* (n = 478)	Q2* (n = 479)	Q3* (n = 479)	Q4* (n = 479)	
**Age, years**	67.5 ± 15.51	68.6 ± 15.15	70.5 ± 14.75	68.3 ± 15.13	62.4 ± 15.86	<0.001†
18–39	102 (5.3)	21 (4.4)	16 (3.3)	22 (4.6)	43 (9.0)	
40–59	443 (23.1)	99 (20.7)	93 (19.4)	101 (21.1)	150 (31.3)	
60–79	864 (45.1)	212 (44.4)	211 (44.1)	232 (48.4)	209 (43.6)	
≥80	506 (26.4)	146 (30.5)	159 (33.2)	124 (25.9)	77 (16.1)	
**Sex**						<0.001†
Female	869 (45.4)	257 (53.8)	274 (57.2)	223 (46.6)	115 (24.0)	
Male	1046 (54.6)	221 (46.2)	205 (42.8)	256 (53.4)	364 (76.0)	
**Race/ethnicity**						<0.001†
White	1164 (60.8)	267 (55.9)	301 (62.8)	312 (65.1)	284 (59.3)	
Black	193 (10.1)	74 (15.5)	51 (10.6)	38 (7.9)	30 (6.3)	
Hispanic/Latino	59 (3.1)	18 (3.8)	11 (2.3)	11 (2.3)	19 (4.0)	
Asian	73 (3.8)	21 (4.4)	17 (3.5)	14 (2.9)	21 (4.4)	
Other	426 (22.2)	98 (20.5)	99 (20.7)	104 (21.7)	125 (26.1)	
**Body mass index, kg/m^2^**	27.6 ± 6.62	27.2 ± 6.49	26.9 ± 7.93	28.2 ± 5.74	28.3 ± 6.03	<0.001†
**Tobacco use**	577 (30.1)	162 (33.9)	124 (25.9)	143 (29.9)	148 (30.9)	0.059
**Use of anticoagulants**	430 (22.5)	145 (30.3)	114 (23.8)	90 (18.8)	81 (16.9)	<0.001†
**Vital sign**						
SBP, mmHg	138.7 ± 23.78	135.9 ± 26.83	142.1 ± 22.81	137.1 ± 21.44	139.8 ± 23.32	<0.001†
DBP, mmHg	75.8 ± 17.35	72.9 ± 18.14	75.7 ± 17.63	76.2 ± 16.22	78.3 ± 16.99	<0.001†
Heart rate, bpm	82.9 ± 17.70	85.0 ± 20.28	83.2 ± 15.72	80.0 ± 16.48	83.2 ± 17.68	<0.001†
Respiratory rate, insp/min	18.6 ± 5.32	18.7 ± 5.40	18.6 ± 5.03	18.4 ± 5.38	18.7 ± 5.49	<0.001†
SpO_2_, %	98 (96-100)	99 (96-100)	99 (96-100)	98 (96-100)	98 (96-99)	<0.001†
Mean arterial pressure	92.8 ± 19.81	90.4 ± 23.63	93.5 ± 18.40	92.5 ± 18.60	94.8 ± 17.87	<0.001†
Body temperature	36.8 ± 0.98	36.8 ± 0.66	36.8 ± 0.65	36.9 ± 0.60	36.8 ± 1.63	<0.001†
**Glasgow Scale Score**	15 (13, 15)	15 (13, 15)	15 (13, 15)	15 (13, 15)	15 (13, 15)	0.609
**Oxford Acute Severity of Illness Score**	33 (27, 39)	35 (28, 42)	34 (28, 40)	32 (26, 38)	31 (24, 37)	<0.001†
**Sequential Organ Failure Score**	3 (2, 5)	4 (2, 6)	3 (2, 5)	3 (2, 4)	2 (1, 4)	<0.001†
**Simplified Acute Physiology Score II**	33 (26, 40)	37 (31, 46)	33 (27, 40)	31 (25, 39)	28 (21, 35)	<0.001†
**Charlson Comorbidity Index**	6 (5, 8)	7.5 (6, 9)	7 (5, 8)	6 (5, 7)	5 (4, 7)	<0.001†
**Comorbidity**						
Diabetes Mellitus	472 (24.6)	151 (31.6)	120 (25.1)	108 (22.5)	93 (19.4)	<0.001†
Chronic Kidney Disease	238 (12.4)	126 (26.4)	59 (12.3)	37 (7.7)	16 (3.3)	<0.001†
Aneurysm	268 (14.0)	69 (14.4)	73 (15.2)	69 (14.4)	57 (11.9)	0.474
Hypertension	773 (40.4)	206 (43.1)	184 (38.4)	194 (40.5)	189 (39.5)	0.492
Atrial fibrillation	432 (22.6)	129 (27.0)	128 (26.7)	99 (20.7)	76 (15.9)	<0.001†
Heart failure	267 (13.9)	115 (24.1)	61 (12.7)	48 (10.0)	43 (9.0)	<0.001†
Coronary heart disease	882 (46.1)	212 (44.4)	246 (51.4)	211 (44.1)	213 (44.5)	0.065
Chronic pulmonary disease	155 (8.1)	50 (10.5)	38 (7.9)	33 (6.9)	34 (7.1)	0.159
Chronic liver disease	148 (7.7)	76 (15.9)	31 (6.5)	19 (4.0)	22 (4.6)	<0.001†
Dementia	110 (5.7)	33 (6.9)	35 (7.3)	30 (6.3)	12 (2.5)	0.005†
**Clinical Laboratory results**						
Glucose, mg/dL	128 (107, 157)	127 (106, 160)	131 (110, 159)	129 (107, 155)	125 (107, 151)	0.304
HbA1c, %	5.7 (5.4, 6.3)	5.7 (5.2, 6.3)	5.7 (5.4, 6.3)	5.8 (5.4, 6.4)	5.6 (5.4, 6.1)	0.141
Ferritin	299.3 (123.5, 622.8)	293 (97.5, 900)	299.5 (147, 654)	257.5 (136, 397.3)	358 (238, 475)	0.784
Serum albumin	3.5 (3.1, 3.9)	3.2 (2.8, 3.6)	3.4 (3.1, 3.8)	3.6 (3.3, 3.9)	3.8 (3.4, 4.2)	<0.001†
WBC, K/uL	10.1 (7.9, 12.9)	9.7 (7.5, 12.8)	9.9 (7.9, 12.9)	10.2 (8.1, 12.6)	10.6 (8.4, 13.5)	<0.001†
RBC, m/uL	4 (3.6, 4.5)	3.3 (2.9, 3.8)	3.8 (3.6, 4.2)	4.2 (3.9, 4.4)	4.5 (4.3, 4.9)	<0.001†
RDW, %	13.8 (13.1, 14.8)	15.8 (14.6, 17.1)	14 (13.5, 14.6)	13.5 (13, 14)	13 (12.5, 13.5)	<0.001†
Platelet count, K/uL	205 (162, 257)	198 (134, 264)	199.5 (158, 255)	209 (168, 256)	210.5 (174, 254)	0.020†
Lymphocyte count, K/uL	1231.7 (820, 1707.5)	1060 (685, 1620)	1151.3 (720, 1590)	1303.5 (930, 1660)	1460 (1010, 1920.	<0.001†
Hemoglobin, g/dL	12.3 (11, 13.5)	9.8 (8.9, 10.7)	11.7 (11.1, 12.3)	12.8 (12.3, 13.4)	14.3 (13.7, 15.1)	<0.001†
BUN, mg/dL	16 (12, 22)	19 (13, 33)	16 (12, 22)	15 (12, 20)	15 (12, 19)	<0.001†
Serum creatinine, mg/dL	0.9 (0.7, 1.1)	1 (0.7, 1.6)	0.9 (0.7, 1.1)	0.8 (0.7, 1.0)	0.9 (0.7, 1.0)	<0.001†
Serum sodium, mEq/L	140 (137, 142)	139 (137, 142)	140 (137, 142)	140 (137, 142)	140 (137, 142)	0.317
Serum Potassium, mEq/L	3.9 (3.6, 4.3)	4 (3.6, 4.4)	3.9 (3.6, 4.2)	3.9 (3.7, 4.3)	3.9 (3.7, 4.2)	0.003†
International Normalized Ratio	1.1 (1.1, 1.3)	1.2 (1.1, 1.4)	1.1 (1.1, 1.2)	1.1 (1.1, 1.2)	1.1 (1.1, 1.2)	<0.001†
CRP, mg/L	14.3 (4.2, 63.3)	39.3 (9.7, 85.2)	27.1 (4.7, 71.7)	7.4 (2.8, 48.4)	8.5 (3, 51)	0.007†
Infection	774 (40.4)	234 (49.0)	218 (45.5)	167 (34.9)	155 (32.4)	<0.001†
ICU LOS	4.1 (2.2, 8.9)	4.5 (2.1, 9.9)	4.4 (2.2, 9.1)	3.9 (2.2, 7.8)	4 (2.2, 8.4)	0.294
Hospital LOS	9 (5, 16)	10 (5, 18)	9 (5, 16)	8 (5, 14)	8 (4, 15)	0.029†
28-d mortality	413 (21.6)	141 (29.5)	106 (22.1)	85 (17.7)	81 (16.9)	<0.001†
1-y mortality	464 (24.2)	163 (34.1)	123 (25.7)	92 (19.2)	86 (18.0)	<0.001†

BUN – blood urea nitrogen, CI – confidence interval, CRP – C-reactive protein, DBP – diastolic blood pressure, HbA1c – haemoglobin A1c, HR – hazard ratio, HRR – haemoglobin/RDW ratio, ICU – intensive care unit, LOS – length of stay, OR – odds ratio, RBC – red blood cell count, RDW – red cell distribution width, SBP – systolic blood pressure, SpO_2_ – saturation of peripheral oxygen, WBC – white blood cell count

*Q1: min ~ 0.7535 (n = 478), Q2: 0.7535 ~ 0.89335 (n = 479), Q3: 0.89335 ~ 1.006845 (n = 479), Q4: 1.006845 ~ max (n = 479).

†Statistically significant results (*P* < 0.05).

Comparison of HRR across the different quartiles indicated statistically significant associations between HRR and most variables listed in **Table 1****.** However, no significant differences were found in tobacco use, GCS score, comorbidities (*i.e.* aneurysm, hypertension, CHD, and COPD) laboratory data (*i.e.* glucose, HbA1c, ferritin, and serum sodium levels), and ICU LOS between the HRR quartiles (**Table 1**).

**Figure 2:**
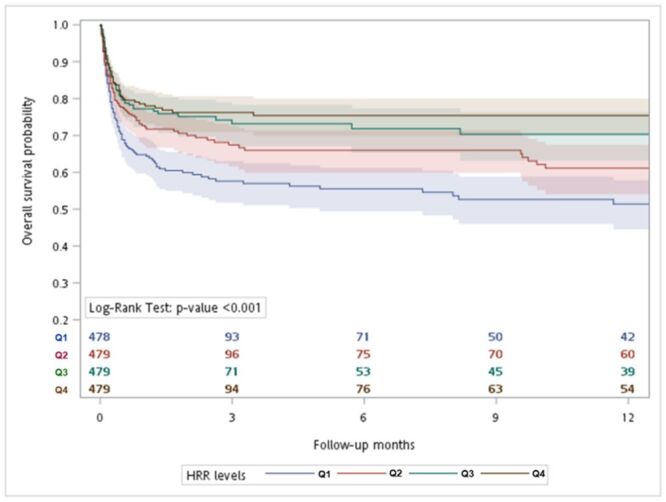
Kaplan-Meier survival analysis plot for 1-year survival probabilities.

Estimated cumulative survival probabilities are illustrated in **Figure 2****.** The *P*-value of the log-rank test was less than 0.00l, revealing statistically significant differences in mortality between the different HRR quartiles. Table S1 in the **Online Supplementary Document** demonstrates the associations between clinical covariates and mortality by univariate analysis. Comorbidities identified through relevant ICD codes are documented in Table S2 in the **Online Supplementary Document**.

### Multicollinearity diagnostics for continuous variables

Pairwise correlation analyses demonstrated no evidence of problematic multicollinearity among the continuous predictors in Table S3 in the **Online Supplementary Document****.** Although several variables showed statistically significant correlations given the large sample size (all *P* < 0.001), the absolute correlation coefficients were modest (|r| values ranging from 0.085 to 0.293). Specifically, HRR showed weak negative correlations with creatinine (r = −0.225), CCI (r = −0.293), and INR (r = −0.204). Creatinine exhibited a weak positive correlation with CCI (r = 0.204) and a negligible correlation with INR (r = 0.085). Similarly, CCI and INR demonstrated only minimal correlation (r = 0.091). All correlation magnitudes were below conventional thresholds indicating multicollinearity (*e.g*. |r|≥0.7), suggesting that these continuous variables can be concurrently included in multivariable models without concern for collinearity.

### Associations between HRR quartiles and mortality

The proportional hazards assumption was evaluated using Schoenfeld residuals. No time-dependent pattern in HRR was observed, whether modelled as a continuous variable (*P* = 0.994 for 28-day mortality; *P* = 0.529 for 1-year mortality) or as quartiles (*P* = 0.302, 0.783 for 28-day mortality; *P* = 0.252, 0.681 for 1-year mortality), indicating that the proportional hazards assumption was satisfied for both short- and long-term mortality analyses.

**Table 2** summarises the associations between HRR quartiles and both short-term (28-day) and long-term (1-year) mortality outcomes. In the unadjusted models, higher HRR quartiles (Q2, Q3, and Q4) were associated with significantly lower risks of 28-day mortality compared to the lowest quartile (Q1) of HRR. Specifically, the hazard ratios (HRs) for Q2, Q3, and Q4 *vs*. those of Q1 were 0.74 (95% CI = 0.58, 0.96), 0.61 (95% CI = 0.47, 0.80), and 0.58 (95% CI = 0.44, 0.77), respectively (all *P* < 0.05). However, after adjusting for potential confounders, multivariable analysis revealed that the associations weakened. The adjusted hazard ratios for Q2, Q3, and Q4 vs Q1 were 0.86 (95% CI = 0.66, 1.12, *P* = 0.27), 0.72 (95% CI = 0.54, 0.97), and 0.67 (95% CI = 0.49, 0.90), respectively.

**Table 2:** Associations between HRR quartiles, 28-d, and 1-y mortality

Outcome/estimate	HRR comparison	Unadjusted		Adjusted*	
		**HR (95% CI)**	***P-*value**	**aHR (95% CI)**	***P-*value**
28-d mortality	Q2 *vs*. Q1	0.74 (0.58, 0.96)	0.021†	0.86 (0.66, 1.12)	0.268
	Q3 *vs*. Q1	0.61 (0.47, 0.80)	<0.001†	0.72 (0.54, 0.97)	0.029†
	Q4 *vs*. Q1	0.58 (0.44, 0.77)	<0.001†	0.67 (0.49, 0.90)	0.009†
1-y mortality	Q2 *vs*. Q1	0.74 (0.58, 0.93)	0.011†	0.79 (0.62, 1.01)	0.061
	Q3 *vs*. Q1	0.58 (0.45, 0.75)	<0.001†	0.64 (0.49, 0.84)	0.001†
	Q4 *vs*. Q1	0.53 (0.41, 0.69)	<0.001†	0.56 (0.42, 0.75)	<0.001†

CCI – Charlson Comorbidity Index, CI – confidence interval, HR – hazard ratio, HRR – haemoglobin/RDW ratio, RDW – red cell distribution width

*Estimates adjusted for use of anticoagulants, CCI, International Normalized Ratio, infection, and creatinine.

†Statistical significance (*P* < 0.05) is shown in bold.

For 1-year mortality, unadjusted models showed that higher HRR quartiles were associated with reduced hazard ratios (HRs) compared to that of Q1. The HRs for Q2, Q3, and Q4 *vs*. Q1 were 0.74 (95% CI = 0.58, 0.93), 0.58 (95% CI = 0.45, 0.75), and 0.53 (95% CI = 0.41, 0.69), respectively (all *P* < 0.01). After adjusting for confounders identified in univariate analysis, the associations remained significant in multivariable analysis. The adjusted HRs for Q3 and Q4 vs those of Q1 were 0.64 (95% CI = 0.49, 0.84, *P* = 0.001) and 0.56 (95% CI = 0.42, 0.75, *P* < 0.001), respectively.

### Subgroup analysis

**Table 3** demonstrates the results of subgroup analyses conducted to explore whether the associations between HRR quartiles and mortality varied based on the presence of CHD or the use of anticoagulant medications. Among patients with CHD, no significant associations were found between HRR quartiles and 28-day mortality, but a significant reduction was observed in 1-year mortality in the highest HRR quartiles (Q4 *vs*. Q1; aHR = 0.62; 95% CI = 0.40, 0.95, *P* = 0.027). In contrast, a trend emerged among patients without CHD. The risks of both 28-day and 1-year mortality were significantly lower in the higher HRR quartiles (Q3 and Q4) compared to the odds of mortality in Q1. Specifically, Q3 *vs*. Q1 had an adjusted HR of 0.59 (95% CI = 0.39, 0.89, *P* = 0.012) for 28-day mortality and an adjusted HR of 0.59 (95% CI = 0.40, 0.87, *P* = 0.008) for 1-year mortality.

**Table 3:** Subgroup analysis of associations between HRR quartiles, 28-d, and 1-y mortality

Stratum	HRR	28-d mortality			1-y mortality	
**aHR (95% CI)**	***P-*value**		**aHR (95% CI)**	***P-*value**
With CHD* (n = 882)	Q2 *vs*. Q1	0.88 (0.59, 1.31)	0.524		0.86 (0.59, 1.23)	0.404
	Q3 *vs*. Q1	0.89 (0.58, 1.36)	0.585		0.80 (0.53, 1.19)	0.269
	Q4 *vs*. Q1	0.68 (0.43, 1.07)	0.093		0.62 (0.40, 0.95)	0.027‡
Without CHD* (n = 1033)	Q2 *vs*. Q1	0.85 (0.59, 1.24)	0.409		0.84 (0.59, 1.20)	0.338
	Q3 *vs*. Q1	0.59 (0.39, 0.89)	0.012‡		0.59 (0.40, 0.87)	0.008‡
	Q4 *vs*. Q1	0.64 (0.42, 0.98)	0.039‡		0.58 (0.38, 0.87)	0.008‡
Anticoagulants user† (n = 430)	Q2 *vs*. Q1	1.11 (0.62, 1.98)	0.724		1.13 (0.65, 1.97)	0.664
	Q3 *vs*. Q1	0.74 (0.37, 1.47)	0.391		0.80 (0.42, 1.53)	0.498
	Q4 *vs*. Q1	1.19 (0.63, 2.25)	0.588		1.17 (0.64, 2.17)	0.607
Anticoagulants non-user† (n = 1485)	Q2 *vs*. Q1	0.84 (0.62, 1.15)	0.277		0.82 (0.62, 1.09)	0.178
	Q3 *vs*. Q1	0.74 (0.53, 1.03)	0.070		0.69 (0.50, 0.93)	0.017‡
	Q4 *vs*. Q1	0.59 (0.41, 0.83)	0.003‡		0.52 (0.38, 0.73)	<0.001‡

aHR – adjusted hazard ratio, CHD – coronary heart disease, CI – confidence interval, HRR – haemoglobin/RDW ratio, RDW – red cell distribution width

*Estimates adjusted for use of anticoagulants, Charlson Comorbidity Index (CCI), International Normalized Ratio (INR), infection, and creatinine.

†Estimates adjusted for the Charlson Comorbidity Index, International Normalized Ratio, infection, and creatinine.

‡Statistical significance (*P* < 0.05) is shown in bold.

When stratified by the use of anticoagulants, no significant associations were observed between the HRR quartiles and short- or long-term mortality. However, in non-users, a stronger association was found between HRR and mortality. Specifically, Q4 *vs*. Q1 was significantly associated with reduced 28-day mortality (aHR = 0.59, 95% CI = 0.41, 0.83, *P* = 0.003) and 1-year mortality (aHR = 0.52; 95% CI = 0.38, 0.73, *P* < 0.001). Q3 *vs*. Q1 was only significantly associated with reduced 1-year mortality (aHR = 0.69; 95% CI = 0.50, 0.93, *P* = 0.017).

### Sensitivity analysis

Table S4 in the **Online Supplementary Document** summarises the prognostic utility of haemoglobin and RDW, the individual components of HRR. Haemoglobin quartiles showed limited prognostic value after multivariable adjustment. Higher haemoglobin was associated with lower unadjusted 28-day and 1-year mortality; however, most adjusted associations were not statistically significant, except for the highest haemoglobin quartile (Q4 *vs*. Q1), which remained inversely associated with 1-year mortality (aHR = 0.71; 95% CI = 0.53, 0.94). In contrast, RDW quartiles demonstrated consistent and graded associations with mortality. Higher RDW was significantly associated with increased 28-day mortality (adjusted HRs for Q2–Q4 *vs*. Q1: 1.44, 1.48, and 1.89) and 1-year mortality (aHRs: 1.41, 1.44, and 1.88).

## DISCUSSION

Results of this large, retrospective cohort study of ICU patients with ICH show that a lower HRR at admission is independently associated with higher risk of both 28-day and 1-year mortality. After adjusting for possible confounders, patients in the higher HRR quartiles (Q3 and Q4) had significantly lower odds of short-term mortality and reduced hazards of long-term mortality compared to those in the lowest HRR quartile (Q1). Subgroup analyses further revealed that the protective association between higher HRR and mortality was more pronounced among patients without CHD and among those not receiving anticoagulant therapy. These findings suggest that HRR, a simple and readily available hematologic parameter, may serve as an independent prognostic biomarker for critically ill patients with ICH.

This study is among the first to evaluate associations between HRR and both short-term (28-day) and long-term (1-year) mortality specifically in critically ill patients with ICH – investigating even further, patients with comorbid CHF and those on anticoagulant therapy. Previous studies did, however, note the utility of HRR in evaluating mortality risk in patients with other types of non-traumatic cerebral haemorrhage or comorbid illness [12,13]. In particular, Liu & Wang (2023), whose study also used the MIMIC-IV database, recently showed that low HRR is associated with increased in-hospital mortality in patients with non-traumatic SAH [12]. Zhong et al. (2024), also using MIMIC-IV data, reported that 30-day and 1-year mortality were reduced in inpatients with sepsis who had higher HRRs on admission [13]. Previous studies have also shown that both of the components of HRR, Hb and RDW, each have prognostic significance in haemorrhagic stroke. Elevated RDW on admission has been identified as an independent predictor of higher mortality in ICH [4,13], and lower haemoglobin levels on admission are associated with greater mortality and disability after ICH [14].

Results of the present study showed that lower HRR is associated with higher mortality in ICH, which aligns with cumulative evidence from previous studies investigating other acute or severe conditions, which also have shown that HRR has prognostic value. For example, a higher HRR at ICU admission was independently associated with improved survival in critically ill sepsis patients [7]. Another study reported that low HRR was associated with higher stroke severity and higher mortality in patients with stroke, concluding that low HRR on admission of patients with stroke is a useful predictive factor for mortality and stroke severity [8].

Regarding the biological mechanisms underlying our findings, anaemia – characterised by lower Hb levels and diminished oxygen transport – can exacerbate brain injury in ICH by reducing oxygen delivery and increasing the susceptibility of the peri-haematomal region to ischemia [15]. A recent study by Law et al. (2024) similarly demonstrated that anaemia and impaired oxygen delivery worsen brain injury in spontaneous intracerebral haemorrhage [16]. In parallel, elevated red cell distribution width (RDW), a marker of anisocytosis and systemic inflammation, has also been associated with adverse outcomes in acute brain haemorrhage. Pinho et al. [4] found that higher RDW at admission independently predicted 30-day mortality in intracerebral haemorrhage. RDW reflects variations in red blood cell size and is increasingly recognised as a surrogate for inflammatory and pathological stress. Supporting this, Akpinar et al. (2021) [17] reported that RDW independently predicted poor functional outcomes in ischemic stroke patients undergoing mechanical thrombectomy. Further, Xie et al. (2024) [18] demonstrated a nonlinear association between HRR and a notably poor prognosis – risk is reduced with low HRR values at admission, suggesting that HRR can be used as a powerful indicator of prognosis for patients with haemorrhagic stroke.

In our subgroup analyses (**Table 3**), the inverse association between higher HRR and mortality was evident in patients without CHD and not receiving anticoagulants, but not in those with CHD or anticoagulant use. Several biological and clinical mechanisms may underlie these differences. First, the absence of a clear association between HRR and mortality in patients with CHD may reflect the fact that CHD is itself characterised by chronic hemodynamic impairment, impaired myocardial oxygen delivery, and persistently elevated RDW driven by inflammation and oxidative stress. RDW is a well-established prognostic marker in coronary artery disease and other cardiovascular conditions, where higher RDW independently predicts adverse events and mortality [19–21]. In this context of chronically abnormal erythrocyte indices, HRR may be less sensitive to the acute physiological insult of ICH, whereas in patients without CHD – who generally have better cardiovascular reserve and lower baseline RDW – acute reductions in HRR may more faithfully capture haemorrhage-related anaemia and inflammatory stress, thereby yielding stronger mortality discrimination. Similarly, anticoagulant users constitute a subgroup in whom prognosis is dominated by hematoma volume, hematoma expansion, and coagulopathy, all of which are strongly linked to anticoagulant-associated ICH and its excess mortality [22,23]. These powerful determinants of outcome may overshadow the more nuanced prognostic information contained in HRR, and reversal strategies can further perturb haemoglobin and RDW over time, diluting HRR’s signal. In contrast, non-anticoagulated patients typically experience a more predictable bleeding profile, allowing HRR to better track the underlying pathophysiology relevant to outcome. Finally, the smaller sizes of the CHD (n = 882) and anticoagulant-treated subgroups (n = 430) and the wide but directionally consistent confidence intervals suggest that part of the null findings may be due to limited statistical power rather than absence of any effect, and raise the possibility of true effect modification that warrants confirmation in larger, dedicated cohorts.

In our sensitivity analysis, haemoglobin alone demonstrated weaker and largely non-significant associations with mortality after multivariable adjustment, indicating that aanemia alone may not sufficiently capture the underlying risk (Table S4 in the **Online Supplementary Document**). RDW alone showed significant associations, consistent with its known link to inflammation and erythropoietic stress, but represents only one dimension of the pathophysiology. In contrast, HRR exhibited (**Table 2**) more robust, consistent, and directionally stable associations across all quartiles and both mortality endpoints, suggesting that integrating oxygen-carrying capacity (haemoglobin) with anisocytosis/inflammatory burden (RDW) provides enhanced prognostic discrimination compared to either component alone. These findings support the biological rationale that HRR captures a broader and more integrated physiologic signal, thereby offering superior prognostic value beyond haemoglobin or RDW individually.

Given its independent association with both short- and long-term mortality in ICU patients with intracerebral haemorrhage, HRR may serve as a clinically actionable biomarker to support early risk stratification at the time of ICU admission. Because HRR is derived from routinely available hematologic indices, it can be readily integrated into existing severity assessment workflows, either as an additive component to established prognostic scores (*e.g*. APACHE II, SOFA) or as a standalone early warning indicator that flags patients requiring heightened monitoring, expedited neuroimaging, or early neurosurgical consultation. In resource-constrained settings, HRR may further inform allocation of ICU-level resources, facilitate triage decisions, and guide discussions regarding prognosis with families. By reflecting both oxygen-carrying capacity and underlying inflammatory burden, HRR provides complementary pathophysiologic insight beyond traditional vital signs and laboratory markers, thereby enhancing its translational value for clinicians managing high-risk ICH patients.

### Strengths and limitations

This study is one of the first investigations to evaluate associations between HRR and both short-term and long-term mortality specifically in critically ill patients with ICH, using a large, well-validated, and publicly available database. The use of the MIMIC-IV database, which contains high-quality, granular ICU data, is a particular strength of this study, enhancing the reliability and generalisability of study findings within critical care settings. The large sample of over 1900 patients provided sufficient statistical power to detect clinically meaningful associations and perform subgroup analyses stratified by important clinical factors such as CHD and anticoagulant use. Also, the study design employed rigorous patient selection criteria, focusing on first ICU admissions during the initial hospitalisation for ICH, and used standardised methods for exposure (HRR) assessment with careful pairing of laboratory measurements, minimising potential misclassification bias. Furthermore, adjustments for a comprehensive set of covariates, including demographic and clinical characteristics, comorbidities, and laboratory parameters, reduced the risk of confounding, supporting the robustness of the study results.

Several limitations must also be acknowledged, including, first, the retrospective, observational study design with only ICU-admitted ICH patients, which limits the applicability of its findings to broader haemorrhagic stroke populations. Because the haemorrhagic stroke cases occur in community and rural settings, the external validity and global relevance of the results are reduced. Also, although we carefully selected the first available Hb and RDW measurements within one day after hospital admission and prioritised values closest to ICU admission, the timing of laboratory measurements may likely have varied between patients, and dynamic changes in HRR over time would not be captured. Besides, HRR quartiles were determined without external validation and clinical consensus, which limits the robust clinical application. Specific causes of death were not available in MIMIC-IV and including these factors in analyses may have influenced outcomes. The present study focused on all-cause mortality without distinguishing specific causes of death (*e.g.* neurological vs systemic), and competing risks (such as non-neurological deaths) which were not available in the database and therefore were not specifically modelled, although this approach is still appropriate for all-cause mortality analysis. Moreover, due to limitation of the database, the confounding factors persist which limited the interpretation for the outcomes, such as nutritional status, socioeconomic factors, prior functional capacity hematoma volume, location, presence of intraventricular haemorrhage, or surgical intervention. Finally, HRR is a composite biomarker influenced by both anaemia and inflammatory processes; thus, while low HRR was associated with poor prognosis, it remains uncertain whether interventions targeting HRR directly would improve clinical outcomes, necessitating further prospective research.

## CONCLUSIONS

A lower HRR at admission is independently associated with higher 28-day and 1-year mortality in critically ill patients with ICH. Study findings suggest that HRR, may serve as a candidate prognostic biomarker for early risk stratification in this high-risk population. Given its accessibility and predictive value, HRR can potentially aid clinical decision-making and resource allocation in intensive care settings. Further prospective studies are warranted to validate these findings and to explore whether dynamic changes in HRR during hospitalisation may offer additional prognostic information.

## Additional material


Online Supplementary Document
