# Supplementary material for: Associations between haemoglobin-to-red cell distribution width ratio and mortality in intracerebral haemorrhage: a population-based analysis of the MIMIC-IV database
Source: J Glob Health. 2026 Apr 17;16:04065. doi: 10.7189/jogh.16.04065 (PMC13088685; doi:10.7189/jogh.16.04065)
Supplement: Online Supplementary Document [file jogh-16-04065-s001.pdf]

Supplement to: Chen BA, Lin SN, Wei KC, Hsieh YY, Yan JL, Chen MY, Kuo YH, Chen YY, Chen PY. Associations between haemoglobin-to-red cell distribution width ratio and mortality in intracerebral haemorrhagenon-traumatic cerebral haemorrhage: a population-based analysis of the MIMIC-IV database. J Glob Health. 2026;16:04065.

Text S1. Explanation of authorship change statement

In the original submission, the author order and corresponding author designation were based on a provisional internal draft and did not fully reflect the contributions of all authors. In particular, additional substantial contributions were made during the revision process, including manuscript improvement and responses to reviewers.

Following further discussion, all co-authors have mutually agreed to revise the author sequence and corresponding author designation to more accurately represent the overall contributions to the work, which resulted in placing Bo-An Chen as the first author and Jiun-Lin Yan as the corresponding author. All authors have reviewed and approved the revised authorship and consent to this change.

We apologise for any inconvenience this correction may cause and appreciate the opportunity to update the authorship to accurately reflect contributions.

STROBE Statement—Checklist of items that should be included in reports of cohort studies

	Item No	Recommendation	Page No
Title and abstract	1	(a) Indicate the study's design with a commonly used term in the title or the abstract	1
		(b) Provide in the abstract an informative and balanced summary of what was done and what was found	2-3
Introduction			
Background/rationale	2	Explain the scientific background and rationale for the investigation being reported	4-5
Objectives	3	State specific objectives, including any prespecified hypotheses	5
Methods			
Study design	4	Present key elements of study design early in the paper	6
Setting	5	Describe the setting, locations, and relevant dates, including periods of recruitment, exposure, follow-up, and data collection	6-10
Participants	6	(a) Give the eligibility criteria, and the sources and methods of selection of participants. Describe methods of follow-up	6-9
		(b) For matched studies, give matching criteria and number of exposed and unexposed	6-9
Variables	7	Clearly define all outcomes, exposures, predictors, potential confounders, and effect modifiers. Give diagnostic criteria, if applicable	7-9, Supplementary Table S2.
Data sources/ measurement	8*	For each variable of interest, give sources of data and details of methods of assessment (measurement). Describe comparability of assessment methods if there is more than one group	6-9

Bias 9 Describe any efforts to address potential sources of bias 7, 9-11
Study size 10 Explain how the study size was arrived at 6-7
Quantitative variables 11 Explain how quantitative variables were handled in the analyses. If applicable, describe which groupings were chosen and why 9-10
Statistical methods 12 (a) Describe all statistical methods, including those used to control for confounding 9-10
(b) Describe any methods used to examine subgroups and interactions 10
(c) Explain how missing data were addressed 7
(d) If applicable, explain how loss to follow-up was addressed 7
(e) Describe any sensitivity analyses 10
Results
Participants 13* (a) Report numbers of individuals at each stage of study—eg numbers potentially eligible, examined for eligibility, confirmed eligible, included in the study, completing follow-up, and analysed 11-12, Figure 1
(b) Give reasons for non-participation at each stage 11-12, Figure 1
(c) Consider use of a flow diagram 11-12, Figure 1
Descriptive data 14* (a) Give characteristics of study participants (eg demographic, clinical, social) and information on exposures and potential confounders 11-12, Table 1
(b) Indicate number of participants with missing data for each variable of interest Figure 1
(c) Summarise follow-up time (eg, average and total amount) 7
Outcome data 15* Report numbers of outcome events or summary measures over time 13-14
Main results
16 (a) Give unadjusted estimates and, if applicable, confounder-adjusted estimates and their precision (eg, 95% confidence interval). Make clear which confounders were adjusted for and why they were included 13-14
(b) Report category boundaries when continuous variables were categorized 13-14
(c) If relevant, consider translating estimates of relative risk into absolute risk for a meaningful time period NA
Other analyses 17 Report other analyses done—eg analyses of subgroups and interactions, and sensitivity analyses 15-16
Discussion
Key results 18 Summarise key results with reference to study objectives 16-17
Limitations 19 Discuss limitations of the study, taking into account sources of potential bias or imprecision. Discuss both direction and magnitude of any potential bias 20-21
Interpretation 20 Give a cautious overall interpretation of results considering objectives, limitations, multiplicity of analyses, results from similar studies, and other relevant evidence 17-21
Generalisability 21 Discuss the generalisability (external validity) of the study results 20-21
Other information
Funding 22 Give the source of funding and the role of the funders for the present

study and, if applicable, for the original study on which the present article is based 22

*Give information separately for exposed and unexposed groups.

Note: An Explanation and Elaboration article discusses each checklist item and gives methodological background and published examples of transparent reporting. The STROBE checklist is best used in conjunction with this article (freely available on the Web sites of PLoS Medicine at http://www.plosmedicine.org/, Annals of Internal Medicine at http://www.annals.org/, and Epidemiology at http://www.epidem.com/). Information on the STROBE Initiative is available at http://www.strobe-statement.org.

Supplementary Table S1. Associations between clinical covariates and mortality: Univariate analysis

**Study variables**	28-day mortality		1-year mortality	
	**OR (95%CI)**	***p-value***	**HR (95%CI)**	***p-value***
Age, years	1.02 (1.01, 1.03)	**<0.001**	1.02 (1.01, 1.03)	**<0.001**
18-39	*ref*		*ref*	
40-59	1.82 (0.93, 3.57)	0.079	1.70 (0.95, 3.03)	0.075
60-79	2.21 (1.16, 4.21)	**0.016**	2.05 (1.17, 3.59)	**0.012**
≥80	3.16 (1.64, 6.09)	**0.001**	2.96 (1.68, 5.23)	**<0.001**
Sex				
Female	*ref*		*ref*	
Male	0.91 (0.73, 1.13)	0.397	0.94 (0.79, 1.13)	0.527
Race/ethnicity				
White	*ref*		*ref*	
Black	1.06 (0.72, 1.56)	0.752	1.01 (0.74, 1.40)	0.936
Hispanic/Latino	1.76 (0.98, 3.14)	0.058	1.39 (0.85, 2.27)	0.193
Asian	1.32 (0.75, 2.31)	0.337	1.31 (0.83, 2.06)	0.250
other	1.76 (1.36, 2.28)	**<0.001**	1.54 (1.25, 1.90)	**<0.001**
Body Mass Index, kg/m^2	1.00 (0.98, 1.03)	0.676	1.00 (0.98, 1.02)	0.817
Tobacco use (Yes vs. No)	0.83 (0.65, 1.06)	0.132	0.82 (0.67, 1.01)	0.067
Use of anticoagulants (Yes vs. No)	0.84 (0.64, 1.10)	0.195	0.74 (0.59, 0.94)	**0.012**
Vital sign				
SBP, mmHg	1.00 (0.99, 1.00)	0.561	1.00 (0.99, 1.00)	0.533
DBP, mmHg	0.99 (0.99, 1.00)	**0.014**	0.99 (0.99, 1.00)	**0.044**
Heart rate, bpm	1.01 (1.00, 1.01)	0.052	1.01 (1.00, 1.01)	**0.021**
Respiratory rate, insp/min	1.02 (1.00, 1.04)	0.063	1.02 (1.00, 1.04)	**0.030**
SpO_2, %	1.02 (0.98, 1.05)	0.330	1.01 (0.98, 1.04)	0.613

Mean arterial pressure	0.99 (0.99, 1.00)	**0.015**	0.99 (0.99, 1.00)	**0.015**
Body temperature	1.04 (0.90, 1.20)	0.590	1.03 (0.92, 1.14)	0.658
Glasgow Scale Score	1.06 (1.00, 1.13)	**0.045**	1.04 (0.99, 1.09)	0.128
Oxford Acute Severity of Illness Score	1.12 (1.10, 1.14)	**<0.001**	1.08 (1.07, 1.09)	**<0.001**
Sequential Organ Failure Score	1.32 (1.27, 1.38)	**<0.001**	1.19 (1.16, 1.22)	**<0.001**
Simplified Acute Physiology Score II	1.08 (1.07, 1.09)	**<0.001**	1.06 (1.05, 1.06)	**<0.001**
Charlson Comorbidity Index	1.08 (1.04, 1.13)	**<0.001**	1.07 (1.04, 1.11)	**<0.001**
Comorbidity				
DM	1.42 (1.12, 1.81)	**0.004**	1.29 (1.06, 1.58)	**0.012**
CKD	1.91 (1.42, 2.57)	**<0.001**	1.61 (1.27, 2.04)	**<0.001**
Aneurysm	1.47 (1.10, 1.97)	**0.010**	1.27 (1.00, 1.61)	0.055
Hypertension	1.00 (0.80, 1.25)	0.974	1.03 (0.85, 1.24)	0.795
Atrial fibrillation	1.44 (1.13, 1.85)	**0.004**	1.34 (1.09, 1.64)	**0.006**
CHF	1.80 (1.35, 2.40)	**<0.001**	1.49 (1.19, 1.88)	**<0.001**
CHD	1.19 (0.95, 1.48)	0.125	1.13 (0.94, 1.35)	0.199
COPD	0.98 (0.66, 1.47)	0.930	0.96 (0.69, 1.34)	0.808
Chronic liver disease	1.78 (1.24, 2.57)	**0.002**	1.58 (1.19, 2.09)	**0.002**
Dementia	1.26 (0.81, 1.96)	0.308	1.35 (0.93, 1.96)	0.111
Laboratory data				
Glucose, mg/dL	1.01 (1.00, 1.01)	**<0.001**	1.00 (1.00, 1.00)	**<0.001**
HbA1c, %	1.03 (0.89, 1.19)	0.690	0.99 (0.88, 1.13)	0.933
Ferritin	1.00 (1.00, 1.00)	0.907	1.00 (1.00, 1.00)	**0.023**
Serum albumin	0.55 (0.43, 0.72)	**<0.001**	0.58 (0.47, 0.72)	**<0.001**
WBC, K/uL	1.04 (1.02, 1.07)	**<0.001**	1.01 (1.00, 1.01)	**0.002**
RBC, m/uL	0.68 (0.58, 0.80)	**<0.001**	0.70 (0.61, 0.80)	**<0.001**
RDW, %	1.19 (1.12, 1.27)	**<0.001**	1.14 (1.09, 1.19)	**<0.001**
Platelet count, K/uL	1.00 (1.00, 1.00)	**<0.001**	1.00 (1.00, 1.00)	**<0.001**
Lymphocyte count, K/uL	1.00 (1.00, 1.00)	0.224	1.00 (1.00, 1.00)	0.202
Hb, g/dL	0.88 (0.84, 0.93)	**<0.001**	0.90 (0.86, 0.94)	**<0.001**
BUN, mg/dL	1.02 (1.02, 1.03)	**<0.001**	1.01 (1.01, 1.02)	**<0.001**
Serum creatinine, mg/dL	1.32 (1.19, 1.45)	**<0.001**	1.12 (1.08, 1.16)	**<0.001**
Serum Sodium, mEq/L	1.03 (1.01, 1.06)	**0.008**	1.04 (1.02, 1.06)	**<0.001**
Serum Potassium, mEq/L	1.24 (1.02, 1.51)	**0.033**	1.23 (1.04, 1.44)	**0.013**
International normalized ratio	1.91 (1.43, 2.56)	**<0.001**	1.45 (1.24, 1.70)	**<0.001**
CRP, mg/L	1.00 (0.99, 1.01)	0.715	1.00 (0.99, 1.01)	0.980

Infection (Yes vs. No)	1.41 (1.13, 1.75)	**0.002**	1.11 (0.92, 1.33)	0.269

Statistical significance(p<0.05) is shown in bold.
Abbreviations: Hb, hemoglobin; HbA1c, Glycosylated hemoglobin; WBC, White Blood Cell Count; RBC, Red Blood Cell Count; RDW, Red Cell Distribution Width; HRR, hemoglobin/RDW ratio; BUN, Blood Urea Nitrogen; ICU, Intensive Care Unit; CHF, Congestive heart failure; CHD, coronary heart disease; COPD, chronic obstructive pulmonary disease; CKD, Chronic kidney disease; CRP, C-Reactive Protein; SpO_2, Saturation of Peripheral Oxygen; SBP, Systolic Blood Pressure; DBP, Diastolic Blood Pressure; OR, odds ratio; HR, hazard ratio, CI, confidence interval.

Supplementary Table S2. International Classification of Diseases code used in the study.

Diagnosis	ICD-9-CM	ICD-10-CM
Non-traumatic cerebral hemorrhage	431	I610, I611, I612, I613, I614, I615, I616, I618, I619
Tobacco use	305.1, V15.82	F17.2, Z72.0, Z87.891
Use of antiplatelet agents/anticoagulants	V58.61	Z79.01
Diabetes Mellitus	250	E10-E13
CKD	403.01, 403.11, 403.91, 404.02, 404.03, 404.12, 404.13, 404.92, 404.93, 582, 583.0-583.7, 585, 586, 588.0	I12.0, I13.1, N03.2-N03.7, N05.2-N05.7, N18, N19, N25.0, Z49.0-Z49.2, Z94.0, Z99.2
Aneurysm	437.3, 430, 442.0, 442.1, 442.3, 442.3, 442.9	I67.1, I60.0-I60.9, I71.0-I71.3, I71.4, I71.8, I71.9
Hypertension	401-405	I10-I16, I1A
Atrial fibrillation	427.31	I48.2, I48.91
CHF	398.91, 402.01, 402.11, 402.91, 404.01, 404.03, 404.11, 404.13, 404.91, 404.93, 425.4-425.9, 428.x	I09.9, I11.0, I13.0, I13.2, I25.5, I42.0, I42.5-I42.9, I43.x, I50.x, P29.0
CHD	410–414	I20-I25
COPD	416.8, 416.9, 491-505, 506.4, 508.1, 508.8	I27.8, I27.9, J40 -J47, J60-J67, J68.4, J70.1, J70.3
Chronic liver disease	070.41, 070.44, 070.51, 070.54, 070.7, 275.01, 291.1, 291.8, 291.9, 303.00, 305.00, 357.0, 425.5, 456.0, 456.1, 456.20, 456.21, 567.23, 571.0, 571.1-571.3, 571.42, 571.5, 571.6, 571.8, 572.2, 572.4, 577.0, 789.5, 980.9	B18.0, B18.2, B19.10, B19.11, B19.2, B19.21, E83.110, K70.3, K72.10, K73.0, K73.2, K73.8, K73.9, K74.3, K74.5, K74.60, K74.69, K75.81, K76.0, K76.6, K76.89
Dementia	290, 294.1, 331.2	F00-F03, F05.1, G30, G31.1

Abbreviations: ICD, International Classification of Disease; CM, Clinical Modification CHF, Congestive heart failure; CHD, coronary heart disease; COPD, chronic obstructive pulmonary disease.

Supplementary Table S3. Multicollinearity Diagnostics for HRR in Short-Term and Long-Term Mortality Models

Variables	HRR	Creatinine	CCI	INR
HRR				
r	-	-0.225	-0.293	-0.204
p	-	<0.001	<0.001	<0.001
Creatinine				
r	-0.225	-	0.204	0.085
p	<0.001	-	<0.001	<0.001
CCI				
r	-0.293	0.204	-	0.091
p	<0.001	<0.001	-	<0.001
INR				
r	-0.204	0.085	0.091	-
p	<0.001	<0.001	<0.001	-

Abbreviations: HRR, hemoglobin/RDW ratio; CCI, Charlson Comorbidity Index; INR, International Normalized Ratio.

Supplementary Table S4. Associations between hemoglobin quartiles, RDW quartiles, 28-day, and 1-year mortality.

Variables/Outcome	Comparison	Unadjusted		Adjusted [a]	
		HR (95% CI)	*p-value*	HR (95% CI)	*p-value*
**Hemoglobin**					
	Q2 vs. Q1	0.61 (0.62, 1.04)	0.099	0.95 (0.72, 1.24)	0.690
28-day mortality	Q3 vs. Q1	0.69 (0.53, 0.90)	**0.006**	0.83 (0.63, 1.10)	0.187
	Q4 vs. Q1	0.66 (0.50, 0.86)	**0.003**	0.77 (0.58, 1.05)	0.099
	Q2 vs. Q1	0.78 (0.62, 1.00)	**0.048**	0.93 (0.72, 1.19)	0.551
1-year mortality	Q3 vs. Q1	0.66 (0.51, 0.84)	**0.001**	0.79 (0.60, 1.02)	**0.072**
	Q4 vs. Q1	0.59 (0.46, 0.77)	**<0.001**	0.71 (0.53, 0.94)	**0.018**
**RDW**					
	Q2 vs. Q1	1.34 (0.99, 1.81)	0.057	1.44 (1.05, 1.98)	**0.024**
28-day mortality	Q3 vs. Q1	1.40 (1.03, 1.91)	**0.030**	1.48 (1.07, 2.05)	**0.017**
	Q4 vs. Q1	2.04 (1.54, 2.71)	**<0.001**	1.89 (1.38, 2.58)	**<0.001**
	Q2 vs. Q1	1.34 (1.01, 1.78)	**0.046**	1.41 (1.05, 1.90)	**0.024**
1-year mortality	Q3 vs. Q1	1.39 (1.04, 1.86)	**0.027**	1.44 (1.06, 1.96)	**0.019**
	Q4 vs. Q1	2.09 (1.60, 2.73)	**<0.001**	1.88 (1.41, 2.53)	**<0.001**

Statistical significance (p <0.05) is shown in bold.

[a] Estimates adjusted for use of anticoagulants, Charlson Comorbidity Index (CCI), International Normalized Ratio, infection, and creatinine.

Abbreviations: CCI, Charlson Comorbidity Index; RDW, Red Cell Distribution Width; HR, hazard ratio; CI, confidence interval.
